# Mental health and psychosocial support for families of missing persons in Sri Lanka: A retrospective cohort study

**DOI:** 10.1186/s13031-020-00266-0

**Published:** 2020-04-06

**Authors:** Ida Andersen, Bhava Poudyal, Arundathi Abeypala, Carla Uriarte, Rodolfo Rossi

**Affiliations:** 1 International Committee of the Red Cross (ICRC), 19 Avenue de la Paix, 1202 Geneva, Switzerland; 2 International Committee of the Red Cross (ICRC), 29 Layards Road, Colombo, 5 Sri Lanka

**Keywords:** Ambiguous loss, ICRC, Mental health, Missing persons, Peer support, Psychosocial support, Sri Lanka

## Abstract

**Background:**

The International Committee of the Red Cross (ICRC) has developed its ‘Accompaniment model’ to address the multi-faceted needs of relatives of people who go missing during armed conflict. In Sri Lanka an Accompaniment Programme was launched in 2015 for the relatives of the more than 16,000 missing persons who remain unaccounted for.

**Method:**

One thousand seven hundred eighty-three relatives who took part in the mental health and psychosocial support (MHPSS) component of the ICRC’s Accompaniment Programme in Sri Lanka between April 2016 and August 2017 were offered eight peer-support group sessions, individual home visits, referrals to local services, and commemoration events to pay tribute to their missing relatives. Symptoms of anxiety and depression (using the HADS scale), somatic difficulties (using the BSI scale) and daily functioning (ICRC scale) were assessed before and after the MHPSS intervention.

**Results:**

Prior to receiving support, Tamil and Muslim ethnicity, ≥60 years of age and civilian status were predictors of severe symptoms of anxiety; Tamil ethnicity, ≥50 years of age and being the mother of a missing person were predictors of severe symptoms of depression; and ≥ 50 years of age and severe symptoms of anxiety and depression were predictors of severe somatic difficulties. After receiving support, the vast majority of the relatives of missing persons showed reduced levels of anxiety (81%), depression (79%) and somatic pain (77%), as well as increased functioning (75%). Predictors of improvement following support were severe levels of distress at baseline and Tamil and Muslim ethnicity. In addition, attending at least three group sessions was a predictor of decreased anxiety, age group 51–60 was a predictor of decreased depression, female gender was a predictor of decreased somatic difficulties, and referrals were a predictor of increased functioning**.**

**Conclusion:**

The MHPSS component of the ICRC’s Accompaniment Programme is a relevant approach to helping families to cope with not knowing the fate and whereabouts of their missing relatives, to reduce distress, to break their social isolation and to resume more functional lives. However, further research is needed, possibly through a controlled trial, to better establish the effectiveness of this approach.

## Background

### ICRC in Sri Lanka

Throughout the world, hundreds of thousands of people go missing as a result of armed conflict or other situations of violence. The relatives left behind can have a multitude of needs. These include the need to know the fate and whereabouts of their missing relative, the need for financial, legal, administrative, psychological and psychosocial support, and the need for recognition and justice.

The International Committee of the Red Cross (ICRC) seeks to address the multi-faceted needs of relatives of missing persons through the Accompaniment model [1]. ‘Accompaniment’ means “walking beside someone” and being supportive whenever necessary. It operates on the premise that families can be helped through empathetic relationships and mutual (i.e. peer) support. Accompaniment aims to address every aspect of the difficulties families face by involving both professionals from a variety of fields and ordinary people from the community. The main goal of accompaniment is to strengthen the ability of individuals and families to deal with difficulties related to the disappearance of their relatives and to resume their social lives. They can do this by making use of their own resources and those available in the community – local and national – and by creating a supportive network.

The ICRC has been present in Sri Lanka since 1989 and has registered more than 34,000 tracing requests [2] relating to persons who went missing during the armed conflict that lasted from 1983 to 2009. Today, around 16,000 of these tracing cases remain open and the families of the missing persons continue to live without knowing the fate and whereabouts of their loved ones. In 2015 the ICRC conducted a family needs assessment [3] and subsequently launched an Accompaniment Programme. So far, the programme has been offered to approximately one third of the families of missing persons registered with the ICRC in Sri Lanka, and it is expected to be offered to all of them by 2022. Alongside the direct support provided to families of missing persons, the programme also works with the relevant authorities at the local and national levels to encourage them to address the multi-faceted needs of the families.

### The need for evidence-based MHPSS interventions

The MHPSS interventions offered to families of missing persons in Sri Lanka were designed on the basis of three main sources of knowledge: first, an assessment of the multi-faceted needs of families of missing persons in Sri Lanka; second, the ICRC handbook on accompanying the families of missing persons, which incorporates the theoretical model of ambiguous loss, [4] taking into account the aggravating circumstances of a post-conflict setting; and third, the ICRC’s experience of implementing similar programmes in other contexts. The family needs assessment highlighted the need for support in coping with the multi-faceted difficulties of having a missing family member, including psychological difficulties. Isuru et al found that having a missing person in the family was a predictor of psychological distress among the family members [5]. Numerous studies from the United States, [6] as well as one study from Nepal, [7] have illustrated the usefulness of incorporating ambiguous loss theory into understanding the MHPSS needs of families of missing persons. The impression among ICRC staff and partners, based on the ICRC’s experience in similar programmes in post-conflict situations in the Balkans, the Caucasus, Nepal and elsewhere, is that such an approach has a very positive impact on the well-being of the relatives of missing persons in general and on their psychological distress in particular. A qualitative evaluation of the Accompaniment Programme in Sri Lanka carried out by an independent consultant in early 2017 [8] confirmed the overall positive impact of the programme and identified several hypotheses to be explored through quantitative methods.

**Fig. 1:**
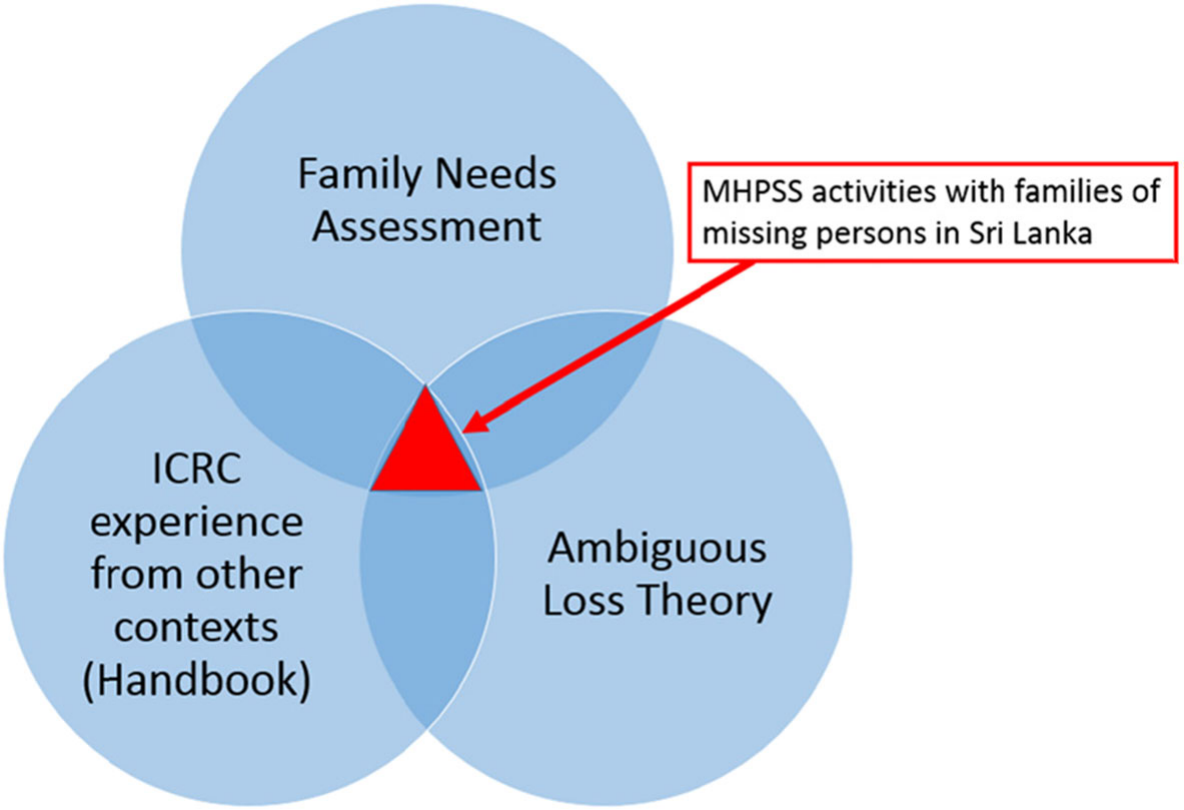
Sources of knowledge on which the ICRC’s activities in Sri Lanka are built

The programme design was founded on a thorough understanding of the MHPSS needs of families, a sound theoretical model and experiences from other contexts, but there is currently no quantitative evidence to support the integration of these three sources of knowledge into MHPSS activities for families of missing persons in Sri Lanka (see red triangle in the figure below) (Fig. 1).

The general lack of evidence-based MHPSS interventions in humanitarian settings has led to the establishment of the MHPSS Research Priority Setting (MHSET) project which, on the basis of key gaps, has identified a consensus-based research agenda covering [9]:

1. the prevalence and burden of mental health and psychosocial difficulties in humanitarian settings,
2. how MHPSS implementation can be improved,
3. evaluation of specific MHPSS interventions,
4. the determinants of mental health and psychological distress, and
5. improved research methods and processes.

This research agenda, along with the ICRC’s growing interest in conducting operational research, led to this study.

### Study goals and objectives

The study aims to better tailor MHPSS interventions to the type and level of mental health and psychosocial needs of the families of missing persons that the ICRC is serving in Sri Lanka. This is achieved by answering a series of research questions. The primary research question is: Does the MHPSS component (see ‘Methodology’ section for more details) of the ICRC’s Accompaniment Programme for families of missing persons in Sri Lanka lead to a decrease in symptoms of anxiety, depression and (psycho) somatic difficulties, as well as an improvement in daily functioning?

A series of secondary research questions explore: first, the differences in improvement depending on the type of support received (e.g. individual or group support, specific sessions and total number of sessions attended); second, the determinants associated with severe suffering of the relatives at baseline such as the characteristics of the *missing person* (e.g. number of years since the person disappeared, age at disappearance and combatant/civilian status) and characteristics of the *relative* (e.g. relationship with the missing person, ethnicity, financial situation and degree of distress prior to the support); third, the determinants associated with improved anxiety, depression, somatic difficulties and functioning following the MHPSS intervention.

## Method

### Study design

The study involves a non-controlled retrospective cohort of 1783 relatives of missing persons in Sri Lanka, who took part in the ICRC’s Accompaniment Programme between April 2016 and August 2017. The data were not initially collected for research purposes but rather as part of the information collected routinely throughout the programme for follow-up and internal monitoring.

### Intervention: the MHPSS component of the ICRC’s accompaniment Programme

Several ICRC departments are involved in addressing the multi-faceted needs of the families of missing persons. The specific mental health and psychosocial support (MHPSS) component is carried out in collaboration with local partner NGOs and selected relatives of missing persons trained to work as accompaniers. All accompaniers are female in order to avoid sending males into female-headed households. They are trained and supported by a multidisciplinary team to understand and address the wide range of needs of the families of missing persons and to provide a bridge to local services [10]. The MHPSS intervention consists of a six-month cycle during which various activities are carried out (Table 1) [11].

**Table 1:** The six-month cycle of MHPSS activities

Month I	Training I of accompaniers on basic communication skills when approaching relatives of missing persons and how to assess multi-faceted needs and make referrals to local service providers
	Collection of household surveys and MHPSS pre-assessments
Month II	
	Training II of accompaniers on conducting peer-support groups on various themes related to the psychological and psychosocial consequences of having a missing relative
Month III-V	Individual home visits and referrals are carried out as needed Eight peer-support group sessions are conducted on the following themes: 1) Introduction and Socialization 2) Our Role in the Family 3) Living with Ambiguity 4) Remembering the Missing 5) Tribute to the Missing (within the group) 6) Commemoration Event (involving the local community) 7) Our Supportive Network 8) Closing
Month VI	Post-psychological assessments are carried out

The MHPSS component of the ICRC’s Accompaniment Programme in Sri Lanka draws heavily on ambiguous loss theory and the notion that uncertainty regarding the fate and whereabouts of a loved one is extremely distressing. This state of perpetual suffering, in which the life of the families of missing persons is put on hold, freezes the natural grieving process and gives rise to feelings of frustration and helplessness. On the social level, this grief can lead to social isolation, a feeling of not being understood by friends or relatives, exclusion and even stigmatization by the community. This suffering, when combined with economic, administrative and other stress-factors, is proven to lead to serious health problems, including anxiety and depression. These manifestations of ambiguous loss are indicative of social and relational problems; they are rooted in external factors which cause stress and ambiguity.

The literature on ambiguous loss purports that the emotional difficulties and manifestations of common mental health problems like anxiety and depression are not to be seen as psychiatric disorders, but rather as a social and relational problem [12]. Thus, the therapeutic goal is to reinvest in emotional attachment, garner social support and build resilience to cope with the ambiguity. The intervention guidelines for working with ambiguous loss [11] were integrated as culturally and contextually adapted themes into eight support-group sessions offered to relatives of missing persons. In addition to conducting group sessions, the ICRC-trained accompaniers also carried out individual home visits and referred families with particular needs (financial, legal, medical or other) to local service-providers under the joint supervision of a coordinator from the partner NGO and an ICRC psychologist.

The accompaniers were selected from the area in which the target families lived and, in most cases, they were themselves relatives of missing persons. The minimum criteria for selection included: high school education, emotional stability (assessed through HADS), mobility (i.e. able to travel around), female gender (to avoid sending males to female-headed households) and motivated to support other relatives of missing persons (assessed through selection interview).

In order to build the capacities of the accompaniers using adult participatory methods, the initial five-day training session included basic support skills (empathic listening and interviewing techniques), how to conduct assessments (i.e. how to use the self-reported questionnaires to assess multi-faceted needs, and the self-reported psychological distress questionnaire), resource mapping (to understand all the resources linked to the different needs of families), understanding and managing stress, and basic techniques of problem-solving-based counselling. Immediately after the initial training session, the accompaniers went to the field to put these skills into practice, under the supervision of the coordinators and the ICRC psychologist. This was followed by a second four-day training session on how to conduct support groups. The training was designed in such a way that the theme-centred group discussions were experienced by the accompaniers first, which allowed them to get first-hand experience of the content of the sessions. To ensure that there was no power dynamic between the accompaniers and the families they supported, the ICRC was in charge of identifying the families to be supported, and the ICRC MHPSS team carried out regular supervision sessions to ensure that the multifaceted needs of all of the families were addressed.

The peer groups consist of eight sessions designed as follows: Session I deals with introductions, setting group norms including confidentiality, constructing a group identity with a group name, and understanding and clarifying the objectives of the support groups. Session II introduces the concept of family system and focuses on helping family members revisit and value the different roles and functions that they have in their lives, while being aware of the roles and functions of other family members. The session aims to facilitate a re-evaluation of the relative’s identity, and thus paves the way for reconstruction. Session III introduces the concept of ambiguous loss and its effect on our mind, our body, our behaviour and our relationships. The session aims to recognize the source of the problems derived from having a missing relative within the social and political context and to normalize the reactions that relatives experience. The session also includes a discussion of the various coping mechanisms that each relative uses within his or her social and cultural context.

Session IV shifts focus away from the relative towards revisiting the memory of the missing person. Each participant shares a personal positive remembrance with the group. At the end of the session, the group members are encouraged to bring something in memory of the missing person (such as an object, food, song, etc.) to the following session to share with the group if they wish to do so. In sessions V and VI, group members are encouraged to discuss ways of paying tribute to their missing relatives as a group. Group members brainstorm ideas and come up with a commemoration event. The commemoration event seeks to allow group members to carry out a meaningful ritual or tribute and gain recognition for their missing relatives, and for themselves. For the actual commemoration event, families invite other community members, including village and/or religious leaders, as well as representatives of authorities where appropriate.

Session VII offers a reflection on the social support network of the group members. This session aims to help them realize that they are not alone and that there are people who are there to help them. A personal map is drawn as a tool for this exercise. Finally, session VIII is a joint evaluation of the group process. The group members are also encouraged to discuss whether they want to continue to meet on their own, and how they would see this through.

### Sources of data

### Data concerning the missing persons whose relatives participated in the accompaniment Programme

1. Tracing request*:* Collected when the relatives approached the ICRC between 1989 and 2009 to register their missing person. The data were collected by an ICRC protection staff member using a closed questionnaire in Sinhala or Tamil when the relative(s) approached the ICRC for registration. The staff member immediately translated and recorded all the information in English in the ICRC Protection database. The following data were used in this study:

- Year of disappearance
- Age at disappearance
- Occupation (combatant or civilian status)
- Ethnicity
- Gender
- Region (north, east or south)

### Data concerning the relatives who participated in the accompaniment Programme

2. Household survey: Collected in 2016 and 2017 when launching the MHPSS activities in a given area, conducted in Sinhala or Tamil by the ICRC-trained accompaniers. Qualitative parts of the household survey were translated into English and entered into the ICRC Protection database by an ICRC data-entry operator. Data used in the study:

- Relationship to the missing person
- Financial status as estimated by the accompanier (extremely poor, poor, moderate, well-off)
- Gender
- Age
- Region (north, east or south)

3. MHPSS pre-, post- and retention assessments: Conducted with all 1783 relatives in 2016 and 2017 prior to the MHPSS interventions (pre-assessment), upon completion 6 months later (post-assessment) and additionally for 285 relatives approximately 1 year after the activities had ended (retention assessment). All of the MHPSS assessments are identical and include the 14-item hospital anxiety and depression scale (HADS), [13, 14] a seven-item, shortened version of the Bradford Somatic Inventory (BSI shortened) as well as a seven-item functioning scale developed by the ICRC using the free listing method. The data were collected in Sinhala or Tamil and, as they were numerical, there was no need for translation prior to entry into Excel. Data used in the study:

- Pre, post and retention HADS scores for anxiety (range 0–7: low: 8–10: mild; 11–14: moderate; and 15–21: severe)
- Pre, post and retention HADS scores for depression
- BSI shortened pre, post and retention scores for somatic difficulties (range 0–7: low; 8–10: mild; 11–14: moderate; and 15–21: severe)
- ICRC functioning scale pre, post and retention scores for daily functioning (rated 0–32 with no standardized cut-off scores) (see scale description in the next section).

### Data concerning the MHPSS intervention

4. Group session attendance sheets: At each of the eight peer-support sessions offered to relatives, attendance sheets were collected by the accompanier and entered into the ICRC Protection database by a data-entry operator. As the data were numerical, there was no need for translation. Data used in the study:

- Attendance of sessions one to eight

5. Individual home visit sheets: After each individual home visit, attendance sheets were collected by the accompanier and entered into the ICRC Protection database by a data-entry operator. Data used in the study:

- Total number of home visits received

6. Referral sheets: Referrals of relatives to local service-providers were recorded by the accompanier and entered into the ICRC Protection database by a data-entry operator. Data used in the study:

- Referred to one or more local service-providers (yes/no)

### Locally developed functioning scale

Given that no scale for measuring daily functioning in the Sri Lankan context currently exists, the ICRC decided to create a local functioning scale using the ‘free listing’ method (Poudyal et al*,* [15]; Bolton et al, [16]). This method consisted of asking 20 adult male and 20 adult female members of families of missing persons, from both the language groups, Sinhala and Tamil, what a “functioning person” in their community would have to be able to do for him- or herself and for others. The sample included a balanced number of both young adults, aged between 21 to 40, and older adults, aged 41 to 60, for both men and women.

The responses from the free listing were compiled in the local languages as a core list and were categorized into the themes that emerged. For example, putting on make-up, cleaning yourself, getting dressed and shaving were all grouped under the theme of “taking care of yourself” with one composite gender-neutral question per theme. A total of seven questions (Table 2) were retained based on the frequency of responses, and the themes they could be grouped under. The questions were put into a five-point Likert scale, from “not at all =0” to “too much = 4” and piloted for comprehension level in both Tamil and Sinhala before the survey was rolled out. The survey results were used to assess the questionnaires’ internal reliability (Cronbach’s alpha). For the Tamil version, the Cronbach’s alpha was 0.87 (*n* = 235), and for the Sinhala version, it was 0.76 (*n* = 127). This local functioning tool has been used within the framework of the Accompaniment Programme for the outcome evaluation of daily functioning.

**Table 2:** ICRC functioning scale for Sri Lanka

In the past two weeks, including today:	None	Little	Moderate	A lot	Too much
1. How much difficulty have you had taking care of yourself?	0	1	2	3	4
2. How much difficulty have you had taking care of your household responsibilities?	0	1	2	3	4
3. How much difficulty have you had concentrating (on any task)?	0	1	2	3	4
4. How much difficulty have you had talking to others?	0	1	2	3	4
5. How much difficulty have you had in taking care of the children in the house?	0	1	2	3	4
6. How much difficulty have you had in visiting friends, neighbours or relatives?	0	1	2	3	4
7. How much difficulty have you had doing other tasks outside of the house?	0	1	2	3	4

### Data management and statistical analysis

All data were numerically coded (categorical data). Quantitative data (i.e. scores, age of missing person, age of disappearance) were kept as such and grouped depending on the type of analysis: either by identifying the median (such as improvement in the various symptoms) and quartiles, or by using established clinical cut-offs (e.g. on the HADS scale from 0 to 21, 15 to 21 indicate severe symptoms of depression).

The dataset was created in Microsoft Excel with two independent data clerks to control for potential typing mistakes. The electronic dataset was protected by a password, which was changed every 3 months. The dataset was transferred to STATA, version 11, for analysis.

All quantitative variables were explored by defining their means (and standard deviation), medians and quartiles. Comparisons of means were tested through the t-test, and the corresponding *p*-value was reported; 95% confidence intervals (95% CI) were calculated around means and means differences. Categorical variables were explored through percentages and tested using the Chi^2^ test to retrieve the corresponding *p*-value; 95% CIs were calculated around these percentages.

To measure associations between variables (crude and multivariable), logistic regression was used to calculate odds ratios (OR) with corresponding 95% CIs and *p*-values from the Wald test. All variables were initially explored in a crude model and were only included in a multivariable model if they were found significant in the crude analysis. Where results from the crude analysis were very similar (within a 10% variation) to the adjusted ones, only the results of the crude analysis were reported to simplify the analysis.

## Results

Of the 2015 relatives who benefited from at least one MHPSS individual- or group-support session between April 2016 and August 2017, complete data was available for 1783 relatives at the time of the study. The discrepancy was primarily due to difficulties in merging data between databases (i.e. between the ICRC Protection database and the ICRC MHPSS scores stored in Excel), as any discrepancy in names due to spelling differences led to mismatches. Most participants were civilians (61.3%), Tamil (80.5%), and female (87.7%). Table 3 presents the demographic and other characteristics of the participants.

**Table 3:** Description of the participants at baseline

Participants characteristics	n	%
**Status of the missing person (*N*** **= 1783)**		
Civilian	1093	61.30
Combatant: government soldier missing in action	217	12.17
Combatant: LTTE	211	11.83
Unknown	262	14.69
**Ethnicity of the missing person (*N*** **= 1783)**		
Sinhala	283	15.87
Tamil	1436	80.54
Muslim	63	3.53
Burger	1	0.06
**Age of the beneficiary (*N*** **= 1417)**		
≤ 40	156	11.01
41–50	279	19.69
51–60	398	28.09
61+	584	41.21
**Sex of the beneficiary (*N*** **= 1783)**		
Male	220	12.34
Female	1563	87.66
**Family relationship (*N*** **= 1783)**		
Mother	815	45.71
Father	169	9.48
Spouse	460	25.80
Siblings	220	12.34
Daughter/son	59	3.31
Other	60	3.37
**Economic status (*N*** **= 1737)**		
Destitute	176	10.13
Poor	890	51.24
Moderate	562	32.35
Well-off	109	6.28
**Region (*N*** **= 1783)**		
North	694	38.92
East	787	44.14
South	302	16.94
**Number of relatives missing (*N*** **= 1783)**		
1	1592	89.29
2+	191	10.71
**Years since disappearance (*N*** **= 1776)**		
≤ 10 years	547	30.80
11–20 years	545	30.69
21+ years	684	38.51
**Another family member also received MHPSS (N = 1783)**		
No	1620	90.86
Yes	163	9.14
**Type of support (*N*** **= 1620)**		
Individual	63	3.89
Group	1557	96.11
**Number of group sessions (*N*** **= 1557)**		
1–2 session(s)	54	3.33
3+ sessions	1503	92.78

### Factors associated with severe distress prior to MHPSS

**Fig. 2:**
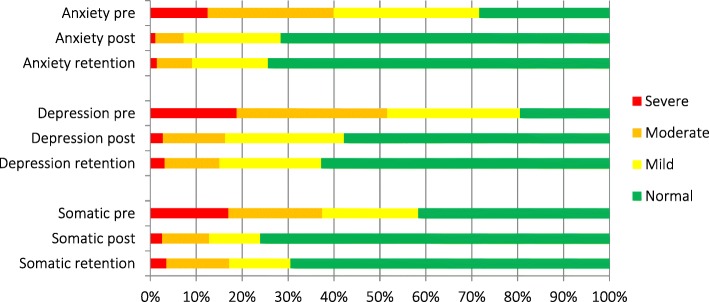
Pre, post and retention severity categories for anxiety, depression and somatic symptoms

At baseline, 12% of the relatives presented severe levels of anxiety; 18% showed severe levels of depressive symptoms; and 16% reported severe distress related to somatic difficulties (Fig. 2). The relatives most likely to present severe symptoms of *anxiety* (defined as having an initial anxiety score above 14) prior to receiving an MHPSS intervention were relatives from the Tamil and Muslim ethnic groups (OR = 2.33 and OR = 2.45, respectively), relatives above 60 years of age (OR = 1.85) and relatives of civilian missing persons, who had 64% higher odds of suffering from severe anxiety compared to relatives of soldiers missing in action. The relatives most likely to present severe symptoms of *depression* (defined as having an initial depression score above 14) prior to receiving an MHPSS intervention were mothers of missing persons, who were likely to have double odds of severity compared to fathers and spouses of missing persons, Tamil relatives (OR = 2.42) and relatives above 50 years of age (OR = 1.81 for those > 50 and OR = 2.46 for those > 60). Relatives of government soldiers missing in action and relatives living in the south of Sri Lanka were significantly less likely to present severe symptoms of depression prior to receiving an MHPSS intervention (OR = 0.40). The relatives most likely to present severe *somatic symptoms* (defined as having an initial somatic score above 14) prior to receiving MHPSS intervention were relatives above 50 years of age (aOR = 2.59 for those > 50 and aOR = 2.41 for those > 60) and relatives who also presented concomitant severe symptoms of anxiety (aOR = 5.03) and depression (aOR = 1.31), both in the crude and multivariable analyses. Gender of the participant was not associated with any of distress factors prior MHPSS (*p* = 0.192 for anxiety, *p* = 0.230 for depression and *p* = 0.201 for somatic symptoms). Factors associated with severe distress prior to MHPSS are presented in Table 4.

**Table 4:** Factors associated with severe distress at baseline

ANXIETY symptoms	OR (95%CI)§	***p***-value*
**Family relationship** (cOR)		
Mother	Ref	–
Father	0.73 (0.42; 1.23)	0.233
Spouse	0.86 (0.61; 1.21)	0.388
Siblings	0.68 (0.42; 1.10)	0.112
Daughter/son	0.69 (0.29; 1.64)	0.400
Other	0.55 (0.22; 1.41)	0.216
**Ethnicity** (cOR)		
Sinhala	Ref	–
Tamil	2.33 (1.41; 3.84)	0.001
Muslim	2.45 (1.05; 5.75)	0.039
**Age of beneficiary (years)** (cOR)		
≤ 40	Ref	–
41–50	1.72 (0.87; 3.42)	0.121
51–60	1.61 (0.83; 3.12)	0.161
61+	1.85 (0.98; 3.49)	0.058
**Status of MP** (cOR)		
Civil	Ref	–
MiA	0.36 (0.20; 0.66)	0.001
LTTE	1.35 (0.91; 1.99)	0.132
Unknown	0.51 (0.31; 0.83)	0.006
**Region** (cOR)		
North	Ref	–
East	0.92 (0.68; 1.24)	0.572
South	0.59 (0.37; 0.93)	0.022
**Years since disappearance** (cOR)		
≤ 10 years	Ref	–
11–20 years	1.07 (0.73; 1.56)	0.737
21+ years	1.14 (0.79; 1.62)	0.486
**Economic status** (aOR)		
Destitute	Ref	–
Poor	1.11 (0.61; 2.01)	0.730
Moderate	0.89 (0.51; 2.65)	0.729
Well-off	1.16 (0.51; 2.65)	0.727
**DEPRESSION**	**OR (95%CI)§**	*p*-value*
**Family relationship** (cOR)		
Mother	Ref	–
Father	0.51 (0.32; 0.80)	0.003
Spouse	0.47 (0.35; 0.64)	< 0.001
Siblings	0.32 (0.20; 0.52)	< 0.001
Daughter/son	0.39 (0.18; 0.88)	0.023
Other	0.51 (0.25; 1.06)	0.073
**Ethnicity** (cOR)		
Sinhala	Ref	–
Tamil	2.42 (1.60; 3.64)	< 0.001
Muslim	0.96 (0.38; 2.42)	0.929
**Age of beneficiary (years)** (cOR)		
≤ 40	Ref	–
41–50	1.49 (0.82; 2.71)	0.192
51–60	1.81 (1.03; 3.18)	0.040
61+	2.46 (1.43; 4.21)	0.001
**Status of MP** (cOR)		
Civil	Ref	–
MiA	0.40 (0.25; 0.65)	< 0.001
LTTE	1.19 (0.84; 1.70)	0.325
Unknown	0.82 (0.57; 1.17)	0.266
**Region** (cOR)		
North	Ref	–
East	0.81 (0.63; 1.05)	0.109
South	0.50 (0.34; 0.73)	< 0.001
**Years since disappearance** (cOR)		
≤ 10 years	Ref	–
11–20 years	1.01 (0.74; 1.38)	0.952
21+ years	1.02 (0.76; 1.38)	0.890
**SOMATIC**	**OR (95%CI)§**	*p*-value*
**Family relationship** (aOR)		
Mother	Ref	–
Father	0.61 (0.44; 1.09)	0.098
Spouse	0.82 (0.55; 1.22)	0.321
Siblings	0.60 (0.32; 1.10)	0.098
Daughter/son	0.48 (0.10;2.26)	0.100
Other	1.50 (0.67;3.37)	0.666
**Age of beneficiary (years)** (aOR)		
≤ 40	Ref	–
41–50	1.61 (0.76;3.42)	0.212
51–60	2.59 (1.26; 5.32)	0.010
61+	2.41 (1.16; 5.00)	0.019
**Status of MP** (aOR)		
Civil	Ref	–
MiA	1.28 (0.74; 2.19)	0.377
LTTE	1.33 (0.84; 2.10)	0.222
Unknown	1.11 (0.72; 1.70)	0.644
**Severe symptoms of anxiety** (aOR)		
No	Ref	–
Yes	5.03 (3.43; 7.39)	< 0.001
**Severe symptoms of depression** (aOR)		
No	Ref	–
Yes	1.31 (1.17; 1.48)	< 0.001
**Years since disappearance** (cOR)		
≤ 10 years	Ref	–
11–20 years	1.14 (0.83; 1.58)	0.423
21+ years	1.03 (0.75; 1.41)	0.855
**Economic status** (cOR)		
Destitute	Ref	–
Poor	0.97 (0.64; 1.47)	0.892
Moderate	0.74 (0.47; 1.56)	0.185
Well-off	0.69 (0.36; 1.34)	0.276

*p-values from Wald Test

§ cOR: Crude OR; aOR: Adjusted OR, only reported if substantially different from the cOR, adjusted for the other variables in the table

### Overall symptom reduction following MHPSS

Following the MHPSS interventions, 81% of the relatives had reduced the severity of *anxiety* symptoms by at least one point and 59% had moved to a lower category of symptoms of anxiety (e.g. from severe to moderate, moderate to mild or mild to normal). Concerning symptoms of *depression*, 79% of the relatives improved by at least one point and 63% had moved to a lower category. As for *somatic* symptoms, 77% of the relatives had improved by at least one point and 49% had moved to a lower category (Table 9 in Appendix). As no standard categories exist for the functioning scale, the percentage of relatives who moved to a lower category could not be calculated.

Among the 285 relatives with whom a retention study was carried out approximately 1 year after an MHPSS intervention, *anxiety* levels were maintained or had further decreased for 61% of the relatives. A maintained level of or further decrease in symptoms of *depression* was seen in 62%, while 38% had maintained or further improved *somatic* symptoms and 47% had maintained or further improved their daily *functioning* (Table 10 in Appendix).

The changes in severity categories of symptoms of anxiety, depression and somatic distress are illustrated in Fig. 2. Functioning is not included in this figure because no standard categories of severity were defined. The vast majority presented normal or mild symptoms following an MHPSS intervention and the improvement was largely maintained over time.

### Factors associated with symptom reduction following MHPSS intervention

#### Anxiety

Relatives most likely to reduce symptoms of anxiety by five points or more (cut-off defined by the median) were: relatives with severe symptoms of anxiety prior to the MHPSS, in a clear linear trend across the categories of severity; relatives who attended three or more group sessions (marginally significant); as well as Tamil and Muslim relatives (adjusted OR = 2.35 for both levels). Relatives of government soldiers missing in action were well over twice as likely to reduce their symptoms (aOR = 2.56). Patients receiving group support were marginally more likely than those receiving individual support to decrease their anxiety symptoms (OR = 1.62). Economic status and time since disappearance were not associated with the likelihood of decreasing anxiety symptoms (data not shown) (Table 5).

**Table 5:** Factors associated with a decrease of at least 5 points in symptoms of anxiety

Variable	OR (95%CI)§	***p***-value*
**Type of support** (cOR)		
Individual	–	–
Group	1.62 (0.95; 2.77)	0.074
**Ethnicity** (aOR)		
Sinhala	Ref	–
Tamil	2.35 (1.26; 4.36)	0.007
Muslim	2.35 (0.97; 5.69)	0.058
**Status** (aOR)		
Civilians	Ref	–
MiA	2.56 (1.31; 5.03)	0.006
LTTE	0.76 (0.53; 1.09)	0.134
Unknown	0.79 (0.57; 1.09)	0.157
**Gender of beneficiary** (cOR)		
M	Ref	
F	1.24 (0.93; 1.66)	0.139
**Age of beneficiary (years)** (cOR)		
≤ 40	Ref	
41–50	1.03 (0.69; 1.54)	0.889
51–60	1.45 (1.00; 2.12)	0.053
61+	1.19 (0.83; 1.71)	0.344
**Family relationship** (cOR)		
Mother	Ref	–
Father	0.85 (0.61; 1.19)	0.355
Spouse	0.89 (0.71; 1.12)	0.334
Siblings	0.72 (0.53; 0.98)	0.038
Daughter/son	1.04 (0.61; 1.76)	0.887
Other	0.71 (0.42; 1.23)	0.222
**Number of group sessions** (cOR)		
Individual (0 sessions)	Ref	–
1–3 session(s)	1.58 (0.81; 3.11)	0.182
4+ sessions	1.63 (0.95; 2.78)	0.074
**Category severity pre-MHPSS** (aOR)		
Normal	Ref	–
Mild	3.41 (2.46; 4.74)	< 0.001
Moderate	10.13 (7.21; 14.24)	< 0.001
Severe	56.13 (32.57; 96.72)	< 0.001

*p-values from Wald Test

§ cOR: Crude OR; aOR: Adjusted OR, only reported if substantially different from the cOR, adjusted for the other variables in the table

#### Depression

Reduction in symptoms of depression by five points or more (cut-off defined by the median) was most likely to occur among relatives with severe symptoms of depression prior to the MHPSS intervention (again, in a clear linear trend) and among relatives aged between 51 and 60 years (OR = 1.60). Again, relatives of government soldiers missing in action were significantly more likely than relatives of missing civilians to decrease their symptoms of depression by five points or more in the multivariable analysis, while this gain was not seen in the crude analysis. Ethnicity and number of sessions attended were not associated with decreased symptoms of depression, neither in the crude (data shown) nor in the adjusted (data not shown) (Table 6).

**Table 6:** Factors associated with a decrease of at least five points in symptoms of depression

Variable	OR (95%CI)§	***p***-value*
**Ethnicity** (cOR)		
Sinhala	Ref	–
Tamil	1.18 (0.91; 1.53)	0.208
Muslim	0.84 (0.48; 1.48)	0.545
**Status** (aOR)		
Civilians	Ref	–
MiA	1.95 (1.29; 2.94)	0.001
LTTE	1.01 (0.71; 1.45)	0.954
Unknown	0.88 (0.64; 1.22)	0.452
**Gender of beneficiary** (cOR)		
M	–	–
F	1.07 (0.81; 1.43)	0.625
**Age of beneficiary (years)** (cOR)		
≤ 40	Ref	–
41–50	1.13 (0.75; 1.69)	0.562
51–60	1.60 (1.09; 2.34)	0.016
61+	1.41 (0.98; 2.04)	0.064
**Family relationship** (aOR)		
Mother	Ref	–
Father	1.31 (0.86; 1.96)	0.212
Spouse	1.17 (0.88; 1.55)	0.284
Siblings	1.60 (1.11; 2.30)	0.012
Daughter/son	2.20 (1.06; 3.77)	0.033
Other	0.79 (0.41; 1.50)	0.467
**Category severity pre-MHPSS** (aOR)		
Normal	Ref	
Mild	4.80 (3.17; 7.26)	< 0.001
Moderate	8.79 (5.84; 13.24)	< 0.001
Severe	52.55 (32.11; 86.02)	< 0.001
**Number of group sessions** (cOR)		
Individual (0 sessions)	Ref	–
1–2 session(s)	1.19 (0.61; 2.33)	0.604
3+ sessions	1.40 (0.83; 2.36)	0.208
**Economic status** (aOR)		
Destitute	Ref	–
Poor	1.18 (.079; 1.77)	0.408
Moderate	0.93 (0.61–1.41)	0.726
Well-off	1.43 (0.81; 2.52)	0.213

*p-values from Wald Test

§ cOR: Crude OR; aOR: Adjusted OR, only reported if substantially different from the cOR, adjusted for the other variables in the table

#### Somatic difficulties

Reduction of somatic difficulties by at least five points (cut-off defined by the median) was most likely to occur in: relatives with severe somatic difficulties prior to the MHPSS activities (in a clear linear trend, as per the other symptoms); Tamil and Muslim relatives (OR = 1.99 and OR = 1.80, respectively); and female relatives (OR = 1.40). Relatives of government soldiers missing in action were significantly less likely than relatives of civilians to show a decrease in somatic symptoms following the MHPSS interventions (OR = 0.56), despite the fact that the somatic symptoms of this group of relatives prior to the MHPSS intervention were not significantly lower than those of the relatives of civilians (Table 6).

#### Functioning

An improvement in functioning of six points or more was most likely to occur among Tamil and Muslim relatives (OR = 7.28 and OR = 4.30, respectively), and relatives who had been referred to local service-providers of various kinds (OR = 1.30). Again, relatives of soldiers missing in action were significantly less likely than relatives of missing civilians (OR = 0.40) to improve their symptoms of functioning by six points or more (Tables 7 and 8).

**Table 7:** Factors associated with an improvement of at least five points in somatic difficulties

Variable	OR (95%CI)§	***p***-value*
**Ethnicity** (cOR)		
Sinhala	Ref	–
Tamil	1.99 (1.52; 2.61)	< 0.001
Muslim	1.80 (1.03; 3.13)	0.038
**Status** (cOR)		
Civilians	Ref	–
MiA	0.56 (0.41; 0.76)	< 0.001
LTTE	1.15 (0.86; 1.55)	0.349
Unknown	0.86 (0.65; 1.13)	0.272
**Gender of beneficiary** (cOR)		
M	Ref	–
F	1.40 (1.05; 1.87)	0.023
**Family relationship** (cOR)		
Mother	Ref	–
Father	0.65 (0.46; 0.91)	0.012
Spouse	0.85 (0.68; 1.08)	0.180
Siblings	0.89 (0.66; 1.19)	0.428
Daughter/son	0.63 (0.37; 1.09)	0.099
Other	1.14 (0.67; 1.92)	0.632
**Category severity pre-MHPSS** (aOR)		
Normal	Ref	–
Mild	6.68 (4.96; 8.24)	< 0.001
Moderate	10.55 (7.79; 14.28)	< 0.001
Severe	24.44 (16.81; 16.82)	< 0.001
**Economic status** (aOR)		
Destitute	Ref	–
Poor	0.95 (0.64; 1.41)	0.782
Moderate	0.82 (0.54; 1.24)	0.236
Well-off	1.27 (0.72; 2.24)	0.409

*p-values from Wald Test

§ cOR: Crude OR; aOR: Adjusted OR, only reported if substantially different from the cOR, adjusted for the other variables in the table

**Table 8:** Factors associated with a decrease of at least six points in difficulties related to functioning

Variable	OR (95%CI)§	***p***-value*
**Ethnicity** (aOR)		
Sinhala	Ref	–
Tamil	7.28 (3.57; 14.83)	< 0.001
Muslim	4.30 (1.71; 10.84)	0.007
**Status** (cOR)		
Civilians	Ref	–
MiA	0.40 (0.29; 0.55)	< 0.001
LTTE	1.26 (0.94; 1.69)	0.125
Unknown	0.90 (0.69; 1.18)	0.441
**Referral** (cOR)		
No	Ref	–
Yes	1.30 (1.02; 1.67)	0.035
**Economic status** (aOR)		
Destitute	Ref	–
Poor	1.26 (0.84; 1.89)	0.261
Moderate	0.95 (0.62; 1.46)	0.830
Well-off	1.01 (0.57; 1.80)	0.979

*p-values from Wald Test

§ cOR: Crude OR; aOR: Adjusted OR, only reported if substantially different from the cOR, adjusted for the other variables in the table

## Discussion

The main research questions have been answered as follows:

I. **Evaluation: Does the MHPSS component of the ICRC Accompaniment Programme for families of missing persons in Sri Lanka lead to a decrease in symptoms of anxiety, depression and (psycho) somatic difficulties, as well as an increase in daily functioning?** Yes. Following the MHPSS interventions, the vast majority of the relatives reduce their psychological distress and increase their daily functioning. This is likely to be explained to a large extent by the fact that the programme offers peer group activities, leading to the creation of what Boss calls *psychological families* [4]*.* As illustrated by the retention scores, these bonds are largely sustained over time and break the isolation that many relatives find themselves in following the disappearance of a family member. Additionally, the MHPSS component is not a set of stand-alone activities, but part of a holistic approach including, among other things, referrals to local service-providers. Many similarities can be found between these characteristics of the MHPSS component of the Accompaniment Programme and the facilitators of successful MHPSS programmes in similar settings identified by Dickson and Bangpan [17]. These include: the development of community partnerships (the network of accompaniers), ongoing training and supervision of MHPSS service-providers (i.e. the accompaniers in this case), meaningful and culturally relevant activities, predominantly group-based activities, and finally the development of trusting and supportive relationships with the MHPSS service-providers (i.e. the accompaniers), who serve as role models.
II. **Determinants: Prior to the support, is there any association between the degree of distress and characteristics of the missing person and/or of the relative?** Yes. Tamil and Muslim relatives, relatives above 60 years of age and relatives of civilian missing persons were most likely to present severe symptoms of *anxiety* at baseline. Tamil relatives, mothers and relatives above 50 years of age were most likely to present severe symptoms of *depression* at baseline. Relatives of government soldiers missing in action and relatives living in the south of Sri Lanka were significantly less likely to present severe symptoms of *depression* prior to the MHPSS intervention. This could be due to the fact that families of government soldiers were systematically informed by the military that their relative had gone missing during combat; death certificates were issued, pensions were provided and there was public acknowledgement of missing soldiers as war heroes. This was not the case for the missing civilians. Finally, relatives above 50 years of age and relatives who also presented severe symptoms of anxiety and depression at baseline were most likely to present severe *somatic* symptoms prior to the MHPSS intervention.
  The financial situation of the relative did not influence the level of distress surrounding the disappearance of a loved one at baseline. This finding is in line linwith Patel et al, [18] who found only a weak association with poverty and income level in developing countries and pointed to other factors to explain greater vulnerability to mental disorders. These factors include the experience of hopelessness and a lack of security, rapid social change and the risks of violence, which, in Sri Lanka, can be said to have impacted all relatives of missing persons to some extent regardless of their level of income. We would add to this list the *social isolation* that relatives of missing persons so often find themselves in.
III. **Determinants: Following the MHPSS intervention, is there any association between improvement and the characteristics of the missing persons, the relatives and/or the MHPSS intervention?** Yes. The main predictor of improvement was severe levels of distress at baseline. Overall, Tamil and Muslim relatives were significantly more likely to improve, whereas relatives of government soldiers were systematically less likely to improve. The fact that Sinhala relatives appeared to improve less could be due to the fact that their anxiety and depression scores at baseline were lower than those of Tamil relatives (i.e. they had less room for improvement), but also to the fact that Sinhala relatives attended fewer group sessions than Tamil relatives. In addition, attendance of three or more group sessions was a predictor of decreased *anxiety*; age group 51–60 was a predictor of decreased *depression*; female gender was a predictor of decreased *somatic* difficulties, although gender was not associated with a higher distress at baseline; and referrals were a predictor of increased *functioning*.

### Strengths and limitations of the study

This study has several strengths. First, it included a large sample size, which made it possible to capture or exclude statistical significance of variables that may have clinical or programmatic implications. Second, the lost to follow-up rate of participants during the follow-up period was not too significant, adding validity to the results. Third, the standardized training provided to the accompaniers is likely to have reduced the interviewer bias – but not the respondent bias.

The main limitation of this study is the absence of a control group created by randomization. Without comparison with an identical group of relatives who did not receive MHPSS interventions, we cannot state with certainty that the changes observed in the relatives were in fact due to the MHPSS they received.

Furthermore, all the data from the MHPSS surveys are self-reported, meaning that it is solely the beneficiary’s own perception of his or her situation that determines the scoring done by the accompanier. This may create an information bias in that some beneficiaries will have a tendency to over- or understate their symptoms. However, as this bias is present in both the pre- and post-MHPSS surveys, any change in symptoms is still measured reliably. Also, the same accompanier conducts the pre/post MHPSS surveys and provides the support, which can be problematic, as some relatives could feel pressured during post-MHPSS survey collection to state that their mental state has improved so as not to appear ungrateful towards the accompanier or make her look bad. Likewise, an information bias may exist at the level of the accompaniers who could wish to report high improvement among beneficiaries so as to appear efficient. However, as pre-post analyses were conducted by the ICRC after the end of the cycles and had no consequences for the accompaniers, they would have no major incentives to manipulate the data collection".

### Implications for the programme

Needs remain high even after many years: Any link between the number of years since the disappearance and the levels of stress at baseline and/or the impact of the support provided to the relative could indicate the ideal timing for the support. However, it appears that beyond 10 years of disappearance – the minimum number of years since disappearance of the missing persons whose relatives were included in this study – timing does not play a significant role. Indeed, the number of years since disappearance did not significantly influence degrees of distress prior to the support or the levels of improvement following the MHPSS activities. This finding is in line with ambiguous loss theory, which states that regardless of the number of years that pass, psychological needs linked to the ambiguity do not necessarily decrease. Thus, whether it is 10, 20 or 30 years since the disappearance, it is never too late to provide meaningful support.

Both close and distant relatives have major MHPSS needs: Although mothers were somewhat more likely than other relatives to present severe symptoms of depression prior to the MHPSS intervention, there was no pattern indicating that close relatives had higher MHPSS needs or responded significantly better to the MHPSS intervention. This suggests that even distant relatives can be highly affected by a disappearance and should continue to be included in MHPSS interventions for relatives of missing persons.

## Conclusion

Clarifying the fate and whereabouts of missing persons is one of the primary needs of the relatives left behind. However, during the often lengthy and complex process of clarification, the relatives’ multi-faceted needs brought on by the disappearance can and should be addressed. MHPSS interventions seem to be most effective when integrated in a holistic response. The inherent objective of MHPSS for families of missing persons is neither to “fix” the psychological problems nor to make them forget and move on. Rather, the aim is to help families cope with not knowing the fate and whereabouts of their missing relatives, reduce distress, break their social isolation and resume more functional lives.

Despite the fact that their family members remain unaccounted for, this study has shown that the vast majority of relatives of missing persons who participated in the MHPSS intervention as part of the ICRC’s Accompaniment Programme in Sri Lanka experienced a decrease in symptoms of anxiety, depression and somatic difficulties as well as an increase in daily functioning. Thus, using peers (accompaniers) in MHPSS interventions seems to be a relevant approach in Sri Lanka. The role of the professionals is shifted towards building the capacities of these peer helpers and supervising their work so that families of missing persons can receive effective care and support that otherwise would not be available to them. Further research is needed, possibly through a controlled trial, to better establish the effectiveness of this approach.

Other areas in which further research is equally needed include contexts with more recent disappearances (less than 10 years), contexts where the conflict is ongoing and, finally, the role of economic support programmes when combined with MHPSS activities for families of missing persons.

## Data Availability

Yes, can be shared upon request.

## Appendix

**Table 9:** Pre and post scores

Scores (***N*** = 1783)	Mean (SD)	95%CI mean	p-value*	Range	Median	n (%) with improvement of at least 1 point
Pre-anxiety score	9.68 (4.19)	9.48; 9.87	< 0.0001	0–21	10	
Post-anxiety score	5.55 (3.57)	5.38; 5.71	< 0.0001	0–21	6	
Difference – anxiety	4.13 (4.45)	3.92; 4.33	< 0.0001	−11 – 19	4	1436 (80.54%)
Pre-depression score	10.76 (4.36)	10.55; 10.96	< 0.0001	0–21	11	
Post-depression score	6.59 (4.11)	6.40; 6.78	< 0.0001	0–21	7	
Difference – depression	4.17 (4.61)	3.96; 4.38	< 0.0001	−13 – 21	4	1415 (79.36%)
Pre-somatic score	9.05 (5.10)	8.81; 9.28	< 0.0001	0–21	9	
Post-somatic score	4.87 (4.17)	4.67; 5.06	< 0.0001	0–21	4	
Difference – somatic	4.18 (5.16)	3.94; 4.42	< 0.0001	−12 – 21	4	1380 (77.40%)
Pre-difficulty in functioning score	9.99 (6.65)	9.68; 10:30	< 0.0001	0–28	10	
Post-difficulty in functioning score	4.91 (5.28)	4.66; 5.15	< 0.0001	0–28	3	
Difference – difficulty in functioning	5.08 (6.34)	4.79; 5.38	< 0.0001	−22 – 26	5	1340 (75.15%)

**p*-value from t-test

**Table 10:** Comparison between post and retention scores

Scores (***N*** = 285)	Mean (SD)	95%CI mean	p-value*	n (%) of relatives with decreased distress
Retention-anxiety score	5.52 (3.80)	5.07; 5.96		
Post-anxiety score	5.41 (3.84)	4.96; 5.86		
Difference – anxiety	0.11 (4.34)	−0.40; 0.61	0.6825	175 (61.40%)
Retention-depression score	7.15 (4.02)	6.68; 7.62		
Post-depression score	6.36 (4.16)	5.88; 6.85		
Difference – depression	0.78 (5.03)	0.20; 1.37	0.0091	177 (62.11%)
Retention-somatic score	5.62 (4.27)	5.12; 6.12		
Post-somatic score	4.88 (3.82)	4.44; 5.33		
Difference – somatic	0.74 (4.76)	0.18; 1.29	0.0094	109 (38.25%)
Retention-difficulty in functioning score	4.91 (5.08)	4.32; 5.50		
Post-difficulty in functioning score	4.21 (4.53)	3.68; 4.74		
Difference – difficulty in functioning	0.70 (5.14)	0.10; 1.30	0.0226	133 (46.67%)

**p*-value from t-test

## Abbreviations

AP: Accompaniment Programme
ICRC: International committee of the red cross
LTTE: Liberation tigers of tamil eelam
MHPSS: Mental health and psychosocial support
MiA: Missing in action (solider of the Sri Lankan army)
